# Analysis of Gambling in the Media Related to Screens: Immersion as a Predictor of Excessive Use?

**DOI:** 10.3390/ijerph15010058

**Published:** 2018-01-02

**Authors:** Jean-Jacques Rémond, Lucia Romo

**Affiliations:** Psychology Department, Laboratory EA CLIPSYD 4430, Paris Nanterre University, 92000 Nanterre, France

**Keywords:** gambling, video-game addiction, screen addiction, immersion, problematic Internet use, comorbidity, cognitive distortion

## Abstract

This study investigates the intricacies between the player interface proposed by the screens, (in particular on smartphone applications or in video games) and gambling. Recent research indicates connections between “immersion” and excessive screen practice. We want to understand the causal-effects between online gambling and the “immersion” variable and understand their relationship and its contingencies. This article empirically investigates whether and how it is possible to observe immersion with its sub-dimensions in gambling on different screens. The objective of this study was to analyze: (1) the costs and benefits associated with gambling practice on screens (2) the link between gambling practice and screen practice (video game, Internet, mobile screen); (3) to observe the propensity to immersion for individuals practicing gambling on screens; and (4) to examine the comorbidities and cognitive factors associated with the practice of gambling on screen. A total of 432 adults (212 men, 220 women), recruited from Ile-de-France (France), responded to a battery of questionnaires. Our study suggests that immersion variables make it possible to understand the cognitive participation of individuals towards screens in general, the practice of gambling on screens and the excessive practice of screens.

## 1. Introduction

This innovative study is an extension of previous studies based on gamblers commitment to screens on slot machine operated electronically [1,2,3,4]. The notion of “gambler” for its part, is understood very broadly and includes any person who would bet on chance [5].

In this study, we consider the practice of the lottery, bingo, sports betting, card games, dice games and electronic slot machines. These practices may include the virtual format.

### 1.1. Gambling

Through the literature, it is easily recognized that these practices have a high participation rate in Western cultures [6,7,8]. The excessive practice of gambling can generate problems that affect all spheres of a person’s life. The negative consequences can be significant and include financial debts, bankruptcy, family disputes and dissolution, criminal behavior and suicidal acts [9,10,11,12,13]. Gambling addiction has recently been reclassified in the DSM-5 [14] in the category of “substance-related addictions and behavioral addictions”. As such, excessive gambling and gambling practice are recognized [15]. Gambling practice exists on a continuum ranging from casual, recreational, problematic and excessive gambling.

Epidemiological research on problematic gambling practice has revealed predominant risk factors, including age, whereby young people are more prone to these risks; living in urban areas than rural areas; socially and economically disadvantaged and with easy access to gambling [16]. Some authors [17,18,19,20] have argued that well-established risk factors are age, gender, cognitive distortions (misperceptions, illusion of control), sensory characteristics, schedules, comorbidities (personality disorder), AD/HD, substance use, depression, anxiety and illegal acts. The difficulty in regulating emotions is also a risk factor. Authors have found that low self-control, emotional dysregulation, a need to change mood, fill an existential or emotional void (lack of social interaction, relieve boredom), need for approval and avoid difficulties, increased the severity of gambling problems [21,22,23].

The distinction in gender composition for the practice of gambling has been particularly studied in the literature [24,25,26,27,28]. The practice of gambling has traditionally been regarded as an essentially masculine activity, but studies have shown a significant prevalence of women [25,26,29]. Men are more likely to play for excitement or sensation seeking, while for women, gambling can be related to emotional regulation. Women also begin gambling practice at a later age than men and generally have a faster rate of gambling than men [30]. The distinction between the reasons for gambling practice between men and women may reflect differential comorbidities between the gender.

Concerning comorbidities, a number of studies have revealed that pathological gamblers are more likely to experience problems of psychiatric comorbidity [9,31,32], among adults and teenagers [33,34,35,36,37,38]. The most common comorbidities found are depression, anxiety, alcoholism and obsessive-compulsive behavior. Studies also report that compulsive gamblers are more likely to report mood disorders, attention deficit disorders with or without hyperactivity [39,40,41,42,43,44]. Clarke found that pathological gamblers were more depressed than gamblers with no gambling problems [45]. This is reflected in recent studies [36]. Pathological gamblers may also experience cognitive distortions, such as denial, superstition, overconfidence, sense of control, etc. [46,47]. Irrational beliefs would be reinforced when the player reaches a high level of immersion while playing on the Internet or on electronic machines [48,49,50,51,52,53].

Even before practicing gambling on modern screens through the Internet, the first interactive mediation with gambling involved electronic slot machines. The first studies on immersion and gambling were based on the interactive format of these machines. They show lower rates of engagement of the general population, in contrast to traditional gambling, but studies show that they are more closely associated with problematic use [48]. Studies describe a gambling trajectory, going as far as problematic practice, more accelerated for players playing on an electronic slot machine (on average one year), in contrast to traditional gambling players (on average three and a half years) [54]. These gambling practices on the screens, within casinos, are also more strongly related to psychological distress than other forms of gambling [55]. This acceleration of short-term commitment to gambling by these media can be explained by the immersive and continuous audiovisual experience that can stimulate the pursuit of the game phases [48,56]. These devices also use a faster play rate compared to other traditional forms of gambling. These variables can be found in gambling formats on the Internet via modern screens.

Individual differences in the tendency to be immersed in gambling via electronic machines can contribute to the development of behaviors towards gambling. Individuals in a state of excessive immersion may no longer be able to perceive external stimuli outside the game [57], future appointments or physiological evidence (such as the need for food or to urinate). Just like the traditional gambling, players report relieving chronic stress or negative emotions [44,57]. If positive reinforcement (earning money) is the motivation, the most common among players, negative reinforcement (avoidance) is a significant predictor of gambling and its severity [58]. An avoidance copying style revolves around leaving or avoiding the stressful situation. Typically, this includes distraction via specific tasks or social engagement. Based on the pathways model, individuals who are emotionally vulnerable endorse an escape pattern of gambling where the primary motivation for gambling is avoidance of negative emotions and situations. Although few empirical studies have observed the “immersion” variable on gambling’s electronic machines, Schull argues that for some players, this mental state of avoidance may be the consequence of gaming behavior on electronic machines [57].

### 1.2. Gambling on Screens

When the mobile game is discussed in research, it was often included under the aegis of “Internet games” [6,59,60,61,62,63], regardless of the different platforms and user behavior. It is essential to distinguish the behavior of a gamer of Internet with elements of gambling according to whether a gamer practices this activity on a fixed screen, or on a mobile screen, or on software dedicated to gambling, or on platforms Video games integrating these gambling forms. Studies have already shown that mobile gambling is associated with a high risk of problem gambling [64].

Gambling software or applications on mobile monitors and screens such as smartphones offer greater interactivity [65,66]. The advent of gambling practice on a smartphone increased the practice of betting and lottery playing [67]. Players now have instant access to their favorite game type. To understand the immersion variable of the gambling player on the mobile screen, it is necessary to understand the playful modality of the media proposed for the player on this screen, as well as the playful mode of the related technology. Numerous studies have examined these two modalities [68,69,70,71].

Gambling on mobile phones includes several ways to access gambling. This can be through an application, an optimized site, a game on the phone, or written messages. The status of these activities as gambling remains uncertain in a regulatory and legislative framework. Gainsbury et al. [59] give an online gambling taxonomy that separates different activities depending on whether the payment is mandatory or optional, depending on luck or skill, the platform on which the game is played and the proposed theme in connection with gambling. In Gainsbury’s taxonomy, the term “online gambling” refers to “gambling on the Internet” where the notions of virtual gambling and gambling are integrated into a gaming format of video games, so the gambler on the Internet will spend the money via the game for a reward and the appearance will be likened to a gambling, but also a wider range of activities such as multiplayer casino video games, practice games, competitions or tournaments based on gambling (for example, poker).

The study also investigate if any model of research on video games on mobile screens can’t get closer to models on gambling when it has concealed a financial incentive and chance. The visual, sound and hedonic appeal would thus could thus to influence the player. Immersion theories largely contribute to explain an individual’s commitment to the screen [72,73,74,75].

### 1.3. Immersion and Gambling

If we consider studying gambling-related immersion, then we have to consider the notion of the player’s commitment and desire to play the game on the screen. Immersion can be defined as “the physical experience of being immersed (...) the sensation of being surrounded by a completely different reality, (…), the whole of our perceptive apparatus” [76]. Immersion also comes from the Latin term “immergo” meaning to be damaged in the sense of a complete absorption that can harm the individual.

The immersion uses the flow principles based on the Csikszentmihalyi theory [77,78]. “Flow is the term given to a constellation of subjective experiences reported by people engaged in fulfilling activities” [72]. The flow experience is multifaceted. First, it involves the attenuation of the different attentional processes; Csikszentmihalyi argues for a definition that would be the fusion of action and sensations, a decrease in the perception of time and a distortion of consciousness [77]. Csikszentmihalyi refers, on several occasions, to flow as a state representing “the highest level of well-being” [77]. However, this assumption is based on the assumption that the activity from which the immersion is derived would be positive or without consequences for excessive use. If gambling activities on screens with their fast pace of play can provide an immersive experience, gamers may unconsciously pursue gambling activities until this results in significant financial losses. If the individual is directed toward a specific goal, evaluative thinking is mitigated during the immersion phase, individuals may be less able to discern whether the accumulated financial losses exceed the expected limits. This conception of immersion related to gambling on screens or on electronic gambling machines has been the subject of few studies. Dixon et al. [79] have shown that the introduction of “concealed losses into victories” in modern media such as electronic machines or screens favors a model of empowering the experience [57]. As it was, the losses still permitted to acquire points of experience or even multiple losses made it possible to obtain all the same credits to replay. Indeed, this function creates a continuous stream of small victories, as opposed to large occasional gains separated by long phases of losses. In the study by Dixon et al. [79], network participants reported an increase in sensation related to immersion. There is a limit to the assessment and interpretation of immersion degree on electronic slot machines. Indeed, few studies assess immersion in relation to excessive practice and propose a holistic model of excessive behaviors related to the cognitive processes in interaction during the phases of viewing and practice of gambling.

For this study, we distinguish the processes of suggestibility to immersion, cognitive processes related to the quality of immersion, as demonstrated by Psotka and Davison [80]. Thus, self-awareness, concentration, attention, self-control are variables belonging to the domain of suggestibility processes of immersion, while sensory skills, the search for new experimentation, persistence of the object, distractibility are processes related to the quality of immersion. A dissociative state could help players cope with stressors and provide relief from aversive states like anxiety, depression or boredom. A number of studies [81,82,83,84,85,86] have shown a link between pathological gambling and the dissociation experiment during gambling phases. Cartmill et al. studied the relationship between anxiety state, dissociation states and gambling problems depending on the severity of gambling problems [81].

Recent studies support the idea that basic attention is disrupted in problem gamblers. Similarly, studies have shown that gambling-related stimuli create interference in the processing of information. In these studies, pathological gamblers had to perform the Stroop test [87,88]. In the Diskin and Hodgins studies, the pathological gambler group responded significantly more slowly to the objectives presented than the casual gamblers. This difference in latency was interpreted as an attentional narrowing resulting from the fact that pathological gamblers overvalued the visual stimuli of the game to the detriment of peripheral events.

In addition to attentional bias and visual processing deficits, studies have shown that players have distractibility [89]. Distractibility can interfere with the processing of affective information, which reduces the empathic analysis of pain stimuli (Kam, Xu, and Handy [90]). It is possible to consider that players with mood disorders pre-existing in the practice of gambling may seek, in the gambling on the screens, a form of escape. These perceptual deficits can cause functional impairment. The elderly present a high risk of idiopathy and report a higher frequency of distractibility. This suggests that distractibility can lead to physiological consequences via a reduction in the processing of environmental information [91].

This study focuses on the immersion process during the phases of cast projection of the screen and gambling stimuli on the screens can be modulated in order to adapt prevention messages during the phases of game on the screens to the gambling players [92], in particular for the new media related to screens that encourage a first gambling experience [93].

## 2. Objectives and Hypothesis

Our study focuses on the link between gambling and the new medias related to screens, with “immersion” as a moderator variable. By examining the costs and degree of gambling practice on the screens, we may presume, on the one hand, the importance of evaluating the practices on the screens as a priority, and on the other hand, to consider evaluating the immersion variable and its sub-dimensions. In order to show the interest of studying the immersion variable, we have to consider that the immersion degree is higher for people at risk of excessive use of screens and even higher for people practicing excessively screens and having a problematic practice of gambling.

### Hypothesis

Following the examination of previous researches in the field, five hypotheses have been formulated:

**Hypothesis** **1 (H1).** *The economic costs of purchasing applications or gambling games on the screens are more important in gamblers with problem gambling than non-problem gamblers*.

**Hypothesis** **2 (H2).** *Individuals with problem gambling practice have higher scores on scales assessing excessive gambling practices*.

**Hypothesis** **3 (H3).** *The propensity to immersion scores are higher: for people with gambling addiction [1], than for those with no problem gambling practice; And for people with gambling addiction and high scores on scales evaluating practice on screens [2]*.

**Hypothesis** **4 (H4).** Individuals with problem gambling and high scores on scales assessing screen practices, unlike those with only a problem gambling practice, have higher scores on scales assessing anxiety and depression; On the scale assessing impulsivity; To scale assessing emotional regulation; On the scale assessing cognitive distortions.

**Hypothesis** **5 (H5).** Individuals with very high scores on the immersion scale (QPI) and low scores at the ICJP scale had significantly lower scores at scales assessing screen practices.

## 3. Methods

### 3.1. Data Collection and Sampling

According to the criteria of the Helsinki Declaration on Consent to Research and Clinical Practice, a paper and online protocol was proposed to a French adult population on a voluntary and anonymous basis. The paper version of the protocol has been distributed mainly to students from universities in Île-de-France region of France (bachelor degree) (*n* = 222; 51.27%) and to those coming from various socio-professional categories (*n* = 211; 48.73%). The online questionnaire was distributed to various social media. No financial incentives were offered. The online questionnaire was filled out on different types of screens (computer, tablet, and smartphone). Four hundred thirty-three questionnaires were collected. The final sample (*n* = 432) comprised 212 men (49%) and 220 women (51%), with an average age of 21.94 years (SD = 5.51).

### 3.2. Scales Related to Games of Gambling

The Canadian Problem Gambling Index (ICJE) is a measure of the severity of gambling problems over the past 6 months. This instrument, developed in English and French, has nine questions rated 0–3 (never, sometimes, most of the time, always). A comparison between the CPGI and another questionnaire commonly used in prevalence studies of pathological gambling, the South Oaks Gambling Screen (SOGS, Lesieur and Blume, [94]) reveals that the two instruments generate identical rates (Ladouceur, Jacques, Chevalier, Sévigny, and Hamel, [95]).

The Gambling Related Cognition Scale (GRCS) is a translated and validated tool of the Gambling Related Cognitions Scale (GRCS). The ECJ has a good psychometric quality and has 23 items on a seven-point Lickert scale that identify a variety of game-related cognitions. It identifies the gambler’s expectations of the game, his control illusions, the predictive power he believes to have on the game, his perception of his ability to resist a desire to play, and the interpretations gained through motivating its continuation of the game.

### 3.3. Immersion

The Questionnaire on the Propensity to Immersion (QPI) (Bouchard et al. [96,97]). The French QPI scale, initially named ITQ, consists of 18 items, on a seven-point Lickert scale, and is a self-questionnaire measuring the propensity for immersion. With a Cronbach α of 0.88, the scale proposes to evaluate the total propensity to immersion and contain four variables (focus, implication, play, and emotion).

### 3.4. Scales Assessing Screen Practices

The Problematic Internet Use Questionnaire (PIUQ) scale has been chosen to evaluate the excessive use of the Internet, validated in French by Kern and Acier [98]). The version used consists of twelve items, on a six-point Lickert scale, and four dimensions (self-control, negative consequences, psychological weaning and Internet concerns). The Cronbach alpha of the overall score is between 0.87 and 0.91 according to the studies of Demrovics, Szeredi and Razsa, [99].

The Smartphone Addiction Scale (SAS) of Kwon et al. [100] was chosen to evaluate smartphone practices. The internal consistency and concurrent validity were analyzed with a Cronbach alpha of 0.97. This scale consists of 34 items on a six-point Lickert scale and was constructed from the Y-scale (scale assessing Internet addiction), a visual analog scale and the diagnostic criteria for abuse and of the psychoactive substance dependence of DSM-IV-TR. A more recent Belgian-French version (Smartphone Addiction Scale Short version or SAS-SV, Psychological Sciences Research Institute, Louvain-la-Neuve, Belgium) (Lopez-Fernandez [101])) exists for adolescents with ten items with a Cronbach alpha of 0.90.

The Video Game Addiction Test was selected to assess the excessive use of video games (Van Rooij et al. [102]). The scale consists of 14 items, on a five-point Lickert scale, where five variables (loss of control, preoccupation, withdrawal symptoms, adjustment/mood modification, conflict) can be visualized. It has good internal consistency with a 0.93 Cronbach alpha.

Additional items were added to the initial protocol to glimpse the different modes of screen-related practices, as well as the financial costs associated with screen practices.

### 3.5. Impulsiveness

“Impulsivity” variable was measured using the UPPS Impulsive Behavior Scale (UPPS, Whiteside, Lynam, Miller and Reynolds, [103]). This evaluation questionnaire was validated in the French version by Van der Linden et al. [104]. The French scale includes 20 items on a four-point Lickert scale and has good psychometric qualities with a Cronbach α of 0.70 to 0.84 for the internal consistency of the different subscales.

### 3.6. Symptomatology

Data on anxiety and depression were collected using Hospital Anxiety and Depression (HAD), a self-rated scale for anxiety-depressive disorders in non-psychiatric populations. The HAD (Zigmond and Snaith, [105]) contains 14 items, divided into two factors (depression: seven items, and anxiety: seven items). The internal consistency varies from 0.68 to 0.93 (mean 0.83) for anxiety, and from 0.67 to 0.90 (mean 0.82) for depression. This scale shows good sensitivity, and specificity for identifying anxiety-depressive disorders.

### 3.7. Emotion

The Emotional Regulation Questionnaire (ERQ) scale was developed by Gross and John [106]). It consists of ten items on a seven-point Lickert scale with two variables (cognitive re-evaluation and cognitive repression). Internal validity has a Cronbach alpha of 0.79 for re-evaluation and 0.73 for cognitive repression.

## 4. Results

Correlations analyses were made between the combinations of variables considered in this study. In order to test the aforementioned hypotheses, statistical analyzes were carried out using the Statistica software (v.13.2, StatSoft society, Tulsa, OK, USA). Descriptive statistics helped to identify the characteristics of the population in our sample. An analysis of variance and correlations (Chi2 and Student’s *t*-tests) were performed. Finally, a multiple regression models and a principal component analysis (PCA) were proposed.

### 4.1. Descriptive Statistics

The average individual in our sample is a French male (*M* = 1.5), aged 22 (*M* = 21.9), with no children (*M* = 0.2), who has at least a bachelor level, and is predominantly student. On average, they spend between 2 and 4 h on the computer, 1–2 h on the tablets, 1–2 h on game consoles and 2–4 h on smartphones. In our sample, 54.04% reported feeling dependent on the computer, 6.7% on tablets, 4.6% on game consoles and 58.66% on smartphones. The costs and wagers according to the gambling practice on the screens were analyzed (Table 1). We also estimated excessive media practices related to screens (Table 2).

Analysis of the correlations on the total sample allowed us to see that the intensity of the gambling practice was positively correlated with the expenses related to the purchase of applications, computer games, video games, and consoles, as well as bets offered on applications, on the computer, and in video games; To the set of cognitive distortions described in the GRCS scale; The intensity of Internet practice (PIUQ); The positive and negative UPPS urgency; The intensity of impulsivity (UPPS); To all the variables making up the intensity of the practice of mobile screens (SAS); To the set of variables of the intensity of the practice of video games; Psychological distress including depression (HAD-D); and to the “game” variable of the immersion scale.

### 4.2. Costs and Prevalence of Gambling/Gambling on Screens

The maximum bet amount differs significantly depending on the intensity of the gambler’s practice. The difference is significant for computerization between non-pathological gamblers and pathological gamblers (*t* = 6.6, *p* < 0.001). The use of mobile and computer screens between non-pathological gamblers and problem gamblers is also significant (*t* = 3.45, *p* = 0.001 for mobile screens and *t* = 3.88, *p* = 0.001). The purchase of computer games is also significant between non-pathological gamblers and problem gamblers (*t* = 3.22; *p* = 0.001). For gambling players on the computer, bets between non-pathological and pathological gambler’s are significantly different (*t* = 2.97, *p* = 0.01) (Table 1). Linked to the screens, players’ bets are also significantly different. Individuals with a problematic practice have higher gambling bets than those with moderate Internet practice (*t* = 2.95; *p* = 0.003). Similarly, those with problem gambling practice are more likely than non-pathologists (*t* = 2.14, *p* = 0.03).

Table 2 illustrates the different practices related to gambling on screens and the intensity of this practice. Anova analysis revealed a significant difference between the different groups representing the intensity of Internet practice (PIUQ) and the score obtained at the ICJP scale (*F* = 23.91, *p* < 0.001). Similarly, we observed a significant difference between the groups representing the intensity of the practice of the mobile screens (SAS) and the scores obtained at the ICJP scale (*F* = 12.27, *p* < 0.001). This significant difference is found in the weighting variables “gambling gamblers on mobile screen applications” (*F* = 11.02, *p* < 0.001), “gambling players on computers” (*F* = 36.24, *p* < 0.001) and “gambling players on mobile, console and computer applications” (*F* = 83.05, *p* < 0.001). Finally, there was a significant difference between the groups representing the intensity of the video games practice and the score obtained at the ICJP scale (*F* = 39.45, *p* < 0.001). The difference is significant only with the weighting variable “players with a gambling practice on mobile screen applications” (*F* = 7.26, *p* = 0.01).

### 4.3. Gambling on Screens and Comorbidities

For gambling on screens, the first elaborated multiple linear regression models consists of 34 variables (*F* = 2.42, *p* < 0.01). These included comorbidities (impulsivity, depression, emotional regulation), cognitive distortions, and scaling variables that evaluated excessive screen-related practices. The variable to be predicted is the practical variable of gambling on the screens with the ICJP scale and the positive variable to play the games of gambling on a screen. This model accounts for 78% of the total variance.

The variable perturbation of daily life (of the SAS scale) (*p* = 0.004) is the best predictor of pathological gambling on screens and especially on mobile screens. Other variables have a predictive value, although lower such as the “lack of premeditation” variable (from the UPPS scale) (*p* = 0.04); The variable “game expectations” (*p* = 0.01) and the “inability to abstain from playing” variable (*p* = 0.01) (from the GRCS scale). The second linear regression model, with 13 closest variables, accounts for 64% of the total variance. The variable “lack of premeditation” (*p* = 0.004), “sensation seeking” (*p* = 0.02), “expectation of the game” (*p* = 0.08), and the total dimensions of the PIUQ (*p* = 0.03) are the predictors of pathological gambling on the screens (Table 3).

In the case of comorbidities (anxiety, depression, emotional regulation, impulsivity, cognitive distortions), the only predictive variable for pathological gambling on the screens was “control illusion” (*p* = 0.03). The 14 variables accounted for 78% of the total variance.

Conversely, the linear regression model suggests that the predictors of excessive use of the Internet shows that 30 variables explain 54% of the total variance, six variables were significant. The variables of gambling practice (*p* = 0.001), tolerance (*p* < 0.001), preoccupation (*p* = 0.003), focus (*p* = 0.01), perturbation of daily life, (*p* = 0.04) and implication (*p* = 0.04) were predictive of excessive use of the Internet.

It is also noted that the predictive variables of excessive video gaming practice when we gamble regularly are different from the excessive practice of pure video gaming practice. For example, the variables “lack of perseverance” (UPPS), “lack of premeditation” (UPPS), the “game” variable of the immersion scale (QPI), the “inability to abstain from playing” GRCS) and “tolerance” (SAS) are predictive of the excessive gambling practice associated with regular gambling (82% of the total variance with 31 variables) (Table 3).

### 4.4. Immersion, Gambling on Screens and Comorbidities

I was observed that immersion scores were significantly higher for people with gambling addiction (Table 4). A Student test was carried out to glimpse the link between immersion and the use of screens. The scores for the VAT, PIUQ and SAS scales are significantly different according to the three immersion thresholds (low, moderate, high), respectively for the PIUQ scale (*t* = 2.51, *p* = 0.01), for the scale SAS (*t* = 3.26, *p* = 0.001) for the VAT scale (*t* = 4.03, *p* = 0.001). The surface analysis of the responses shows that, starting from a high immersion threshold, the individual is less likely to have a high score on scales assessing the intensity of Internet practice and on the scale evaluating the intensity of gambling practice.

Multiple linear regression analysis was used to analize the predictive power of immersion for the practice of gambling on screens in relation with comorbidities. In our model, 14 variables were integrated which explained 48% of the variance (*F* = 2.84, *p* = 0.004) with impulsivity, emotional regulation, anxious and depressive comorbidities and cognitive distortions. Predictors of immersion in the practice of games of chance on the screens are positive urgency (UPPS), anxiety (HAD), predictive power (GRCS), inability to Refraining from Playing (GRCS) and Interpretation for Game Continuation (GRCS).

A principal component analysis (PCA) was carried out to study the relationship between immersion according to the intensity of the gambling practice on the screens and the other variables of our study. Three axes were selected, representing 52.92% of the total variance. The first axis (λ = 26.51%) is characterized and particularly correlated, on the negative side, by all cognitive distortions (GRCS); By the variables implication, emotion, game and total dimensions of immersion; The practice of mobile displays (SAS); The Negative Emergency and the Positive Urgency of Impulsivity (UPPS), and Internet Practice (PIUQ). This axis is a “size” factor, ordering individuals according to their scores on all scales. The two axis (λ = 14.98%) is characterized by anxiety (HAD), all variables of immersion on the positive side and the inability to abstain from playing (GRCS) Impulsivity (UPPS), including sensation seeking (UPPS) and negative urgency (UPPS). The three-axis (λ = 11.43%) is characterized by negative urgency (UPPS), positive urgency (UPPS) and sensation seeking (UPPS), total impulsivity (UPPS) and the illusion of control and interpretation favorable to the pursuit of the game on the negative side.

## 5. Discussion

The objective of this study was to assess the practice of gambling on screens, and to understand the impact of the perceived intensity of immersion by an individual on the practice of screens and gambling on the screens, as well as determining the associated comorbidities. The results obtained in this study highlight certain psychological and psychopathological specificities of gamblers on screens related to immersion.

First, the prevalence of problematic practices is consistent with the literature, particularly for Internet practice (2.27% versus 1–3.2% in the literature for European countries including France and Germany) [107,108,109], particularly for the practice of gambling (i.e., 1.85% in our study against 0.2–3% according to the estimate of the prevalence studies in France) [107,108,109,110], for the practice of video games (13.35% for our study against 14% for the most recent French study) and for the practice of mobile screens (2.77% lower for our study unlike the study Validation of the SAS scale which was at 9.6%) (Tablets, smartphones) [111].

The results of our study confirmed that pathological and problematic gamblers are more prone to spend more money than non-pathological gamblers and also significantly different depending on the interface chosen, whether it is a fixed screen or mobile, via a console or a computer. These increased costs can be explained by the ease of access, the possibility of betting during uninterrupted periods and the immersive interactivity of gambling software on the Internet [3,112,113,114]. Our first hypothesis is confirmed.

We also showed that individuals who were tending to excessive gambling had significantly higher scores on scales assessing fixed-screen and mobile screens (whose behaviors had been assessed by a recent scale that focused solely on self-action directed to a mobile display, including the smartphone or touchpad) (Table 2). The practice of gambling could thus participate in the propensity of excessive practices related to screens. It is important, however, to see the same cognitive distortions found in these different types of excessive practices [115,116]. The intensity of immersion would therefore be a predominant factor in the detection of the excessive practice related to the screen. This confirms the high level of commitment generated during excessive practice phases, as well as the role of the dissociation contained in the “focus” variable [117,118].

Our fourth hypothesis is only partially confirmed. Concerning, the practice of gambling on mobile screens, the only cognitive distortion variable, “predictive power” was significantly different according to the three groups. Regarding only the practice of computer gambling, the set of cognitive distortion scores, with the exception of gambling expectations, were significantly different between the groups. Concerning the practice of gambling on all the screens combined, the variable cognitive distortion was not significant. However, depressive variables (HAD) and lack of perseverance (UPPS) were significantly different across groups. Although the literature demonstrates that cognitive distortions can predict future commitment to excessive gambling, gaming expectations differ according to the interface used, and cognitive distortions are much less pronounced in practice on mobile screens [47,119,120].

The analysis of the area of responses between three variables (PIUQ, ICJP and QPI) showed that, starting from a high immersion threshold, the individual is less likely to have a high score at the scales evaluating the “Intensity of Internet and gambling practice”. Immersion could thus be perceived as a protection factor from a certain threshold.

Finally, variables explaining pathological gambling on the screens allow us to adjust the prevention modalities for the gambling on the screens. Indeed, taking into account the format of the screen (for mobile or fixed purposes), daily life disruption (SAS) variables, lack of premeditation (UPPS), sensation seeking (GRCS), expectations related to the (GRCS) and impulsivity (total PIUQ sums) were predictive of excessive gambling.

There are limitations to this study. The research was constructed from self-assessment, dealing with general behavioral behavior with respect to screens and the Internet and not only on immersion experienced during a main task (a task that would be delimited by a specific action on a continuum within a virtual universe of gambling). This may be a bias in assessing the prevalence of excessive screen-related practices in this population. The average age of our population is also low, our sample could have been enlarged by older people. Also, we could have chosen scales assessing anxiety-depression disorders regularly used in clinical practice such as “Generalized Anxiety Disorder scale (GAD-7) or Patient Health Questionnaire (PHQ). Finally, the distribution of practices according to their intensities is heterogeneous. Better distribution of groups would have been preferable. Neurophysiological measurements and longitudinal analysis would allow a more precise glimpse of the cognitive processes related to immersion variables.

## 6. Conclusions

This study explored the involvement of gambling gamblers in the screens. It also showed the implication of the immersion variable in relation to the intensity of the practice of gambling on the screens. Immersion thus plays a mediating role between cognitive resources and the impact on screen practices.

The practice of gambling begins in adolescence. Studies also indicate that those who gamble during childhood will be more likely to become compulsive gamblers later in life [85,121,122]. It would therefore be essential to reinforce our understanding of the negative consequences of gambling-related behaviors on the screens and also to increase the methods of prevention and intervention [123].

The interest is also to be able to modulate the variables of immersion in the therapeutic management of excessive practice or, upstream, to improve prevention by proposing prevention messages at precise level thresholds Immersion according to the variables composing it [124]. It is also a possibility for game developers to modulate cognitive factors related to immersion leading to excessive practice and thus avoid future problems of excessive and impulsive behavior.

## Figures and Tables

**Table 1:** Costs and prevalence of gambling.

Cost Buying by Material	Total (*n* = 432)		ICJP NPb (*n* = 397) (A)		ICJP Ar (*n* = 19) (B)		CPGI Path (*n* = 8) (C)		*T* *p* A vs. B	*T* *p* A vs. C	*T* *p* B vs. C
	*M* (€)	SD (€)									
(Total sample, Mean) Mobile Application Cost buying Bets	*n* = 56, 12.93%		*n* = 37, 9.32%		*n* = 5, 26.32%		*n* = 1, 12.5%		0.27	0.33	0.93
	12.23	38.04	12.95	40.92	9	8.94	2	/	0.78	0.73	0.37
	*n* = 35, 8.08%		*n* = 31, 7.81%		*n* = 8, 42.11%		/		3.45	0.27	0.51
	0.91	3.74	1.03	3.96	15.5	35.36	/	/	<0.001	0.78	0.62
(Total sample, Mean) Computer Cost buying Bets	*n* = 58, 13.39%		*n* = 40, 10.08%		*n* = 6, 31.58%		*n* = 2, 25%		3.21	1.75	0.23
	55.31	88.07	45.12	60.74	113.33	192.94	85	91.92	0.001	0.08	0.82
	*n* = 34, 7.85%		*n* = 29, 7.3%		*n* = 4, 21.05		*n* = 1, 12.5%		3.88	6.6	0.21
	21.03	86.1	3.1	9.58	143.75	239.21	50	/	<0.001	<0.001	0.84
(Total sample, Mean) Game console Cost buying Bets	*n* = 34, 7.85%		*n* = 22, 5.54%		*n* = 3, 15.79%		*n* = 2, 0.46%		1.66	0.43	1.21
	118.33	141.79	112.5	142.59	200	180.28	60	56.57	0.1	0.66	0.24
	*n* = 19, 4.39%		*n* = 16, 4.03%		/		/				
	1.05	3.15	1.25	3.42	/	/	/	/			

CPGI: Canadian Pathological Game Index, (NPb): Non-problematic; (Ar): At risk; (Path): Pathological.

**Table 2:** Scores of the intensity of the practices on the screens (PIUQ, SAS, VAT) for gambling players (ICJP).

Scales	ICJP NPb (*n* = 397)		ICJP Ar (*n* = 19)		ICJP Path (*n* = 8)		Anova 1		Anova 2	
PIUQ practices	total sample (*n*)	Mean (%)	*n*	*M* (%)	*n*	*M* (%)				
PIUQ NP	131	33	/		/					
PIUQ a little	239	62.2	10	52.63	3	37.5				
PIUQ Prob	27	6.8	9	47.37	5	62.5				
PIUQ PrSign	9	2.27	/		/					
	M.	ET.	M.	ET.	M.	ET.	*F*	*p*	*F*	*p*
PIUQ Tot	28.93	9.99	41.16	7.68	44.63	5.53	21.1	0.001	11.9	0.001
AC	2.89	1.17	3.44	1.01	4	0.56	5.41	0.004	4.28	0.02
CN	2.59	0.99	3.58	0.77	3.63	0.98	13.05	0.001	7.59	0.001
SP	2.09	1.1	3.47	1.25	3.5	0.76	20.34	0.001	5.8	0.005
P	2.11	0.89	2.89	0.99	3.75	0.64	19.81	0.001	6.92	0.002
DT	2.41	0.83	3.35	0.64	3.72	0.46	21.04	0.001	11.99	0.001
SAS practices	Total Sample (*n*)	Mean (%)	Total Sample (*n*)	Mean (%)	Total Sample (*n*)	Mean (%)				
SAS NP	392	98.74	16	84.21	6	75				
SAS Dp	11	2.77	2	10.53	2	25				
SAS Abus	3	0.76	1	5.26	/					
	M.	ET.	M.	ET.	M.	ET.	*F*	*p*	*F*	*p*
SAS Tot	69.43	28.69	90.26	32.55	103.88	25.52	10.04	0.001	5.45	0.007
PVQ	9.46	4.59	12.42	5.29	16.13	4.52	11.57	0.001	8.49	0.001
AP	15.98	7.82	20.26	9.1	23.38	7.19	5.97	0.002	2.8	0.07
RS	12.15	6.15	15.21	6.36	19.38	3.89	7.49	0.001	2.98	0.05
ROC	13.63	6.48	17.68	8.23	21.25	5.7	8.52	0.001	3.12	0.05
SU	10.75	4.97	14.11	5.04	12.75	3.58	4.7	0.009	4.13	0.02
T	7.45	4.24	10.58	4.27	11	4.78	7.41	0.001	3.32	0.04
VAT practices	Total Sample (*n*)	Mean (%)	Total Sample (*n*)	Mean (%)	Total Sample (*n*)	Mean (%)				
VAT NP	353	88.92	8	42.11	3	37.5				
VAT Path	53	13.35	11	57.89	5	62.5				
	M.	ET.	M.	ET.	M.	ET.	*F*	*p*	*F*	*p*
VAT Tot	23.11	11.43	36	10.37	35.38	15.95	15.49	0.001	3.87	0.03
PC	7.28	3.81	10.89	3.41	9.88	5.25	9.65	0.001	5.2	0.009
Pré	4.81	2.67	7.47	2.74	7.75	3.81	13.13	0.001	2.3	0.11
SS	1.69	1.11	2.47	1.43	2.63	1.6	6.85	0.001	0.65	0.65
AMH	3.14	1.8	5.11	2.08	5.75	2.66	17.89	0.001	1.09	0.34
C	6.19	3.41	10.05	3.24	9.38	4.72	14.51	0.001	5.44	0.007

Table 2: CPGI: Canadian Pathological Game Index, (NPb): Non-problematic; (Ar): At risk; (Path): Pathological; PIUQ: Problematic Internet Use Questionnaire, (NP): Non pathological; (Little): little risk; Prob: Problems; PrSign: significant problem; (SC): Self-checking; (NC): Negative consequences; (PW): Psychological weaning; (C): concern; (TD): Total dimension. SAS: Smartphone Addiction Scale, (NP): Non-pathological; (PD): Probable dependence; (Abuse): excessive abuse with repercussions on daily life; (DDL): Disruption of daily life; (PA): Positive Anticipation; (WS): Withdrawal Social; (COR): Cyberspace-oriented relationship; (OU): Over-use; (T): Tolerance. VAT: Video Game Addiction Test, (NP): Non-pathological; (Path): Pathological; (LoC): Loss of Control; (C): Concern; (WS): Weaning syndrome; (AMM): Adaptation/Modification of Mood; (C): Conflicts. Anvoa 2 with variable control “gambling players playing on at least one of the three screen types” (smartphone/touch pad, computer, console). The significance threshold is the threshold *p* < 0.05.

**Table 3:** Comorbidities and gambling on screens.

Scales and Sub-Dimensions	CPGI NPb (*n* = 397)		CPGI Ar (*n* = 19)		CPGI Path (*n* = 8)		Anova 1 Traditional Gambling		Anova 2 Gambling on Console and Mobile Screen		Anova 3 Gambling on Computer		Anova 4 Gambling on All Media Together	
	M.	SD.	M.	SD.	M.	SD.	*F*	*p*	*F*	*p*	*F*	*p*	*F*	*p*
							2.07	0.001	2.37	0.33	2.04	0.006	53.21	0.02
GRCS	43.61	26.08	50.75	33.69	93.5	12.5	4.07	0.02	3.98	0.14	6.51	0.003	1.11	0.37
- ALJ	8.67	5.23	10.75	7.44	14.75	3.77	1.41	0.25	7.1	0.07	2.54	0.09	1.47	0.28
- IC	6.13	4.05	18.5	3.87	5.38	4.69	8.32	0.001	0.85	0.5	17.12	0.001	1.35	0.31
- PP	12.43	8.5	13.38	8.21	26	3.74	4.3	0.01	15.32	0.02	4.94	0.01	1.1	0.37
- IA	9.02	6.77	12.13	9.4	16.75	5.38	2.46	0.09	3.05	0.19	2.61	0.08	1.51	0.27
- IF	7.35	4.73	9.13	7.4	17.5	1.91	5.64	0.003	3.22	0.18	7.56	0.001	1.43	0.29
UPPS	46.76	8.9	51.75	11.34	/		0.02	0.89	2.34	0.24	1.04	0.36	2.94	0.1
- UN	9.46	3.42	10.63	2.88	/		0.22	0.64	1.52	0.35	0.48	0.62	0.45	12.82
- UP	11.26	3.01	11	2.45	/		1.5	0.22	0.52	0.64	0.22	0.8	1.31	0.32
- RS	11.37	2.82	12	2.73	/		0.64	0.43	5.75	0.09	0.79	0.46	1.51	0.27
- MPer	7.22	2.8	9.38	2.67	/		5.06	0.03	1.97	0.28	2.21	0.13	6.17	0.02
- MPré	7.46	2.36	8.75	2.71	/		4.08	0.04	1.96	0.29	1.48	0.24	7.88	1.17
ERQ	4.28	1.11	4.13	1.1	4.93	0.74	1.39	0.25	5.2	0.11	0.76	0.47	0.3	0.75
- RC	4.34	1.42	4.29	1.5	4.96	0.94	1.96	0.14	3.41	0.17	0.37	0.69	0.1	0.91
- RE	4.21	1.24	3.88	1.19	4.88	1.09	0.35	0.7	8.78	0.06	0.89	0.42	0.88	0.17
HAD	11.23	5.4	10.2	1.08	16.5	8	4.09	0.02	32.03	0.009	3.82	0.03	3.67	0.07
Anxiety	6.54	3.38	5	3.21	9	3.37	0.29	0.75	5.55	0.09	1.91	0.16	1.83	0.21
Depression	4.02	2.92	3	1.6	7.75	6.24	0.82	0.44	8.07	0.06	3.36	0.04	5.08	0.03

CPGI: Canadian Pathological Game Index, (NPb): Non-problematic; (Ar): At risk; (Path): Pathological; HAD: Hospital Anxiety and Depression; GRCS: Gambling Related Cognition Scale; IF: Interpretation Favorable; IC: Illusion of Control; PP: Predictive power; ERG: Expectations Related to the Game; AI: Inability to Stop Playing; ERQ: Emotion Relationship Questionnaire; CR: Cognitive reassessment; ER: Expressive repression; UPPS: Impulsive Behavior Scale; NU: Negative urgency; PE: Positive Emergency; SS: Sensation Seeking; LPe: Lack of perseverance; LPr: Lack of premedication. The Anova for the UPPS concerned only problematic and non-pathological gamblers. Anova 2 with control of the variable “practice of gambling on console and mobile screens”. Anova 3 with control of the variable “practice of gambling on a computer”. Anova 4 with control of the variable “practice of gambling all confused screens”. The significance threshold is the threshold *p* < 0.05.

**Table 4:** Scores related to propensity to immersion according to the intensity of gambling alone/gambling on screens.

Immersion Variables	CPGI NPb (*n* = 397)		CPGI Ar (*n* = 19)		CPGI Path (*n* = 8)		Anova 1 Traditional Gambling		Anova 2 Gambling on Console and Mobile Screen		Anova 3 Gambling on Computer		Anova 4 Gambling on All Media Together	
	M.	SD.	M.	SD.	M.	SD.	*F*	*p*	*F*	*p*	*F*	*p*	*F*	*p*
Immersion (QPI)	53.46	18.22	55.16	19.17	63.5	11.98	2.26	0.01	34.84	0.001	24.85	0.001	80.10	0.001
Focus	17.88	6.53	16.37	6.61	18.38	3.54	0.52	0.59	21.47	0.001	45.65	0.001	26.86	0.001
Implication	14.66	6.58	15.37	6.19	17	3.63	0.6	0.55	12.5	0.001	10.85	0.001	31.53	0.001
Emotion	11.96	5.84	11.47	4.34	14.13	3.36	0.63	0.53	23.69	0.001	25.98	0.001	26.45	0.001
Game	5.61	4.23	8.47	4.25	10.25	2.87	8.68	0.001	286.94	0.001	142.33	0.001	760.52	0.001

CPGI: Canadian Pathological Game Index, (NPb): Non-problematic; (Ar): At risk; (Path): Pathological; QPI: Immersion propensity questionnaire. Anova 2 with control of the variable “Intensity of practices on the Internet”. Anova 3 with control of the variable “Intensity of the practices of video games”. Anova 4 with control of the variable “Intensity of practices on mobile screens”. The significance threshold is the threshold *p* < 0.05.
